# Predictive factors of metamorphopsia after reduced-fluence photodynamic therapy in patients with central serous chorioretinopathy with good baseline visual acuity

**DOI:** 10.1371/journal.pone.0240557

**Published:** 2020-10-12

**Authors:** Mayuka Hayashida, Akiko Miki, Shunichiro Nakai, Wataru Matsumiya, Hisanori Imai, Sentaro Kusuhara, Makoto Nakamura

**Affiliations:** Division of Ophthalmology, Department of Surgery, Kobe University Graduate School of Medicine, Kobe, Japan; Medical Research Foundation (Sankara Nethralaya), INDIA

## Abstract

This retrospective study was conducted to investigate the predictive factors associated with metamorphopsia after reduced-fluence photodynamic therapy (RFPDT) in patients with central serous chorioretinopathy (CSC) with good baseline visual acuity. A total of 36 eyes of 36 consecutive patients with resolved CSC after RFPDT and best-corrected visual acuity (BCVA) better than 1.0 (logarithm of the minimal angle of resolution (logMAR) 0) at baseline were examined. Metamorphopsia was measured using M-CHARTS at 12 months after RFPDT. An average of the horizontal and vertical M-CHARTS scores was applied for defining the extent of metamorphopsia. The association between M-CHARTS score at 12 months after RFPDT and clinical parameters (age, sex, duration of symptoms, BCVA, and findings of optical coherence tomography (OCT)) was investigated at baseline or 12 months after RFPDT. The M-CHARTS score at 12 months correlated significantly with duration of symptoms (*P* = 0.005), baseline outer nuclear layer (ONL) thickness (*P* = 0.009), central foveal thickness (CFT) (*P* = 0.001) at 12 months, and ONL thickness (*P* = 0.001) at 12 months after RFPDT. In the multivariate analysis of baseline-related factors, thinner ONL thickness before RFPDT (*P* = 0.010) was significantly associated with large metamorphopsia at 12 months after RFPDT in CSC patients with good baseline BCVA. Baseline ONL thickness may be a useful predictive factor of metamorphopsia after RFPDT in CSC patients with good baseline BCVA.

## Introduction

Central serous chorioretinopathy (CSC) is characterized by localized detachment of the neurosensory retina at the macula, predominantly in middle-aged men [1, 2]. The presenting symptoms in patients with CSC are minor vision blurring, central scotoma, and metamorphopsia [1, 2]. Acute CSC often resolves spontaneously within a few months without visual impairment, whereas patients with chronic and recurrent CSC require treatment [1, 2]. Reduced-fluence photodynamic therapy (RFPDT) with various modifications of standard photodynamic therapy (PDT) is reportedly effective for complete resolution of subretinal detachment (SRD) in patients with either acute or chronic CSC [3–5]. However, no treatment strategy exists for patients with chronic and recurrent CSC with good baseline visual acuity. When treating patients with CSC, improving their visual acuity and symptoms, including metamorphopsia, is essential.

Fujita et al. [6] investigated metamorphopsia before and after RFPDT in patients with CSC using the M-CHARTS score and reported that both vertical and horizontal M-CHARTS scores significantly improved in patients with good baseline visual acuity, whereas there was no improvement in the scores in patients without good baseline visual acuity. However, in clinical practice, it is observed that patients with good baseline visual acuity often exhibit metamorphopsia despite the complete resolution of SRD. Therefore, designing a treatment intervention to avoid metamorphopsia that disturbs the quality of vision is necessary.

Till date, there is no clear evidence regarding the appropriate timing for the treatment of patients with CSC with good baseline visual acuity. Therefore, we conducted this study to investigate the predictive factors of metamorphopsia after RFPDT in patients with CSC with good baseline visual acuity.

## Materials and methods

The protocol of this study adhered to the tenets of the Declaration of Helsinki and was approved by the institutional review board of Kobe University Hospital.

We retrospectively reviewed a total of 36 eyes of 36 consecutive patients with resolved CSC after RFPDT who were treated between March 2016 and April 2019 at Kobe University Hospital and had best-corrected visual acuity (BCVA) ≥1.0 (logarithm of the minimal angle of resolution (logMAR) 0) at baseline. The inclusion criteria were (1) presence of subretinal fluid involving the fovea in optical coherence tomography (OCT) images, (2) presence of angiographic leakage caused by CSC detected on fluorescein angiography, (3) presence of abnormally dilated choroidal vessels detected on indocyanine green angiography, and (4) at least 12-month follow-up after RFPDT. The exclusion criteria were (1) severely myopic or hyperopic eyes with refractive errors (spherical equivalent) of more than six diopters; (2) evidence of choroidal neovascularization; (3) presence of any other ocular disease that could affect visual acuity, including tilted disc syndrome, dome-shaped macula, and uveitis; (4) history of laser photocoagulation, transpupillary thermotherapy, or anti-vascular endothelial growth factor treatment; and (5) presence of media opacities, such as cataracts, that could interfere in acquiring high-quality OCT, fluorescein angiography, and indocyanine green angiography images.

Clinical data regarding age, sex, and duration of symptoms were collected. At baseline, all patients underwent a complete ophthalmologic examination, including slit-lamp examination, dilated fundus examination, and BCVA measurement. The Spectralis OCT system (Heidelberg Spectralis OCT; Heidelberg Engineering GmbH, Heidelberg, Germany) was used for obtaining macular scans and infrared reflectance (IR) images. The Spectralis OCT system (Heidelberg Spectralis HRA2; Heidelberg Engineering GmbH) was used for digital fluorescein and indocyanine green angiographies. The clinical data recorded in this study included M-CHARTS score at 12 months, OCT parameters, and BCVA at baseline and 12 months after RFPDT.

For Spectralis OCT examinations, the average values were obtained for horizontal and vertical line scans through the fovea. At baseline, the central foveal thickness (CFT) was measured from the inner surface of the neurosensory retina to the outer surface of retinal pigment epithelium (RPE) at the fovea. The SRD height was defined as the distance between the outer surface of the sensory retina and the inner surface of RPE at the fovea. The outer nuclear layer (ONL) thickness was measured as the distance between the outer surface of the inner limiting membrane and the inner surface of the external limiting membrane (ELM) at the central fovea. The elongated photoreceptor outer segment (POS) length was defined as the distance between the ELM and the tip of the elongated POS. The increase in ONL thickness was defined as the amount of ONL thickness increased at 12 months after RFPDT compared to that at baseline. The area of SRD was calculated by plotting the SRD lesion on the IR images based on a previous report [7]. We also evaluated the presence of pigment epithelial detachment (PED), the disruption of the ELM layer, and the disruption of the ellipsoid zone (EZ) layer within 500 μm from the fovea. Eyes in which these findings were detected in at least one scan of horizontal and vertical line scans were classified as having the presence of these findings. Since evaluating the disruption of the EZ layer at baseline was sometimes difficult because of the SRD or POS, we evaluated it only at 12 months after RFPDT.

The degree of metamorphopsia was evaluated using M-CHARTS. The angular separation of the dots ranged from 0.2° to 2.0° in this study, and the examination distance was 30 cm; the eye refraction distance was adjusted to this distance. M-CHARTS scores were determined for both vertical and horizontal lines. An average of the horizontal and vertical M-CHARTS scores was applied to define the extent of metamorphopsia. We did not investigate M-CHARTS before RFPDT because we were unable to assess some patients using M-CHARTS because of central scotoma. The presence of metamorphopsia was defined as a mean M-CHARTS score of ≥0.5° at 12 months after RFPDT according to previous reports [8, 9].

All patients received an infusion of verteporfin (Visudyne; Novartis, Basel, Switzerland) at 6 mg/m^2^ body surface area over 10 min; laser treatment was administered 15 min after the initiation of infusion. The standard light intensity was 600 mW/cm^2^, and the irradiation time was shortened to 42 s (half-time PDT). The spot size covered the areas with active leaking spots on fluorescein angiography images.

The decimal visual acuity was converted into logMAR units for statistical analyses. The differences in logMAR BCVA and OCT findings between before and 12 months after RFPDT were analyzed using the Wilcoxon signed-rank test. Statistical comparisons between the two groups were performed using Fisher’s exact test for categorical variables. Spearman’s rank correlation test was used to analyze the association between M-CHARTS score at 12 months after RFPDT and clinical parameters at baseline or 12 months after RFPDT. A multiple regression analysis was conducted to identify the independent baseline parameters associated with metamorphopsia outcome. The differences in ONL findings between the two groups based on the presence or absence of metamorphopsia were tested using the Mann–Whitney U test. A P value <0.05 was considered statistically significant. Statistical analyses were performed using the SPSS software, version 24.0 (IBM Corp., Armonk, NY, USA).

## Results

A total of 36 eyes of 36 patients were included in this study. Table 1 shows the clinical characteristics of the patients. The average duration of symptoms was 7.8 ± 13.3 months. After RFPDT, the BCVA (−0.163 ± 0.063) was significantly improved at 12 months compared to that at baseline (−0.085 ± 0.070) (*P* < 0.001) (Table 2). CFT was significantly decreased at 12 months compared to that at baseline (*P* < 0.001). On the other hand, the ONL was significantly increased at 12 months compared to that at baseline (*P* < 0.001). All eyes exhibited complete resolution of SRD, and two eyes had PED at 12 months after RFPDT.

**Table 1:** Clinical characteristics.

Case number, n	36
Age, years	51.5 ± 10.3
Sex (male/female)	28/8
Duration of symptoms, months	7.8 ± 13.3
PDT spot size, μm	3964.7 ± 1103.1

PDT, photodynamic therapy

**Table 2:** Changes in visual acuity and optical coherence tomography parameters at baseline and 12 months after reduced-fluence photodynamic therapy.

	Baseline	12months	*P* value
BCVA, logMAR	−0.085 ± 0.070	−0.163 ± 0.063	<0.001*
M-CHARTS score, °	-	0.51 ± 0.61	-
CFT, μm	371.3 ± 116.3	203.7 ± 31.1	<0.001*
Height of SRD, μm	211.1 ± 110.7	-	-
Area of SRD, μm^2^	8.4 ± 8.3	-	-
ONL thickness, μm	64.4 ± 15.2	75.8 ± 21.6	<0.001*
Elongated POS length, μm	94.8 ± 37.3	-	-
Presence of PED (+/−)	10/26	2/34	0.071**
Disruption of ELM layer (+/−)	36/0	10/26	-
Disruption of EZ layer (+/−)	-	11/25	-

BCVA, best-corrected visual acuity; logMAR, logarithm of the minimum angle of resolution; CFT, central foveal thickness; SRD, serous retinal detachment; ONL, outer nuclear layer; POS, photoreceptor outer segment; PED, pigment epithelial detachment; ELM, external limiting membrane; EZ, ellipsoid zone.

* Wilcoxon signed-rank test

** Fisher’s exact test.

We analyzed the correlation between the M-CHARTS score at 12 months and the clinical parameters at baseline. Our results showed that the duration of symptoms and ONL thickness correlated significantly with the M-CHARTS score at 12 months (*P* = 0.005 and 0.009, respectively) (Table 3). Regarding the correlation between the M-CHARTS score at 12 months and the clinical parameters at 12 months, the CFT, ONL thickness, and the increase in ONL thickness showed significant correlations with the M-CHARTS score at 12 months (*P* = 0.001, 0.001, and 0.021 respectively) (Table 4).

**Table 3:** Correlation between M-CHARTS score at 12 months after reduced-fluence photodynamic therapy and clinical parameters at baseline.

	*r*	*P* value*
Age, years	0.167	0.329
Duration of symptoms, months	0.457	0.005
PDT Spot size, μm	0.112	0.528
BCVA, logMAR	−0.100	0.561
CFT, μm	−0.212	0.214
Height of SRD, μm	−0.236	0.167
Area of SRD, μm^2^	−0.021	0.901
ONL thickness, μm	−0.431	0.009
Elongated POS length, μm	0.142	0.409
Presence of PED (+/−)	0.166	0.334
Disruption of ELM layer (+/−)	-	-

PDT, photodynamic therapy; BCVA, best-corrected visual acuity; logMAR, logarithm of the minimum angle of resolution; CFT, central foveal thickness; SRD, serous retinal detachment; ONL, outer nuclear layer; POS, photoreceptor outer segment; PED, pigment epithelial detachment; ELM, external limiting membrane.

* Spearman’s rank correlation coefficient.

**Table 4:** Correlation between M-CHARTS score at 12 months after reduced-fluence photodynamic therapy and clinical parameters at the same time point.

	*r*	*P* value*
BCVA, logMAR	−0.011	0.948
CFT, μm	−0.518	0.001
ONL thickness, μm	−0.523	0.001
Increase in ONL thickness, μm	−0.383	0.021
Presence of PED (+/−)	−0.035	0.838
Disrupted ELM layer (+/−)	0.000	1.000
Disruption of EZ layer (+/−)	−0.261	0.125

BCVA, best-corrected visual acuity; logMAR, logarithm of the minimum angle of resolution; CFT, central foveal thickness; ONL, outer nuclear layer; PED, pigment epithelial detachment; ELM, external limiting membrane; EZ, ellipsoid zone.

* Spearman’s rank correlation coefficient.

A multiple regression analysis was conducted to investigate the baseline factors associated with the M-CHARTS score at 12 months after RFPDT. Results showed that a thinner ONL was significantly associated with greater metamorphopsia at 12 months after RFPDT in patients with CSC with good baseline visual acuity (*P* = 0.010) (Table 5).

**Table 5:** Baseline factors related to M-CHARTS score at 12 months after reduced-fluence photodynamic therapy.

	Coefficient	Standard error	*P* value*
Duration of symptoms, months	0.178	0.007	0.260
ONL thickness at baseline, μm	−0.423	0.006	0.010

ONL, outer nuclear layer.

* Multiple regression analysis.

We next evaluated the ONL thickness with or without metamorphopsia after RFPDT. The average baseline ONL thickness values in patients with or without metamorphopsia after RFPDT were 56.3 ± 8.8 and 69.0 ± 16.3 μm, respectively, showing a significant difference between groups (*P* = 0.008) (Table 6).

**Table 6:** Outer nuclear layer thickness with or without metamorphopsia at 12 months.

Metamorphopsia	(+) n = 13	(−) n = 23	P value*
ONL thickness at baseline, μm	56.3 ± 8.8	69.0 ± 16.3	0.008
Increase in ONL thickness, μm	7.2 ± 10.7	13.8 ± 12.6	0.123
ONL thickness at 12 months, μm	63.5 ± 11.9	82.7 ± 22.9	0.002

ONL, outer nuclear layer.

* Mann–Whitney *U* test.

Finally, we performed receiver-operating characteristic (ROC) curve analysis to determine the optimal cut-off value for the presence or absence of metamorphopsia at 12 months after initial treatment. ROC curve analysis demonstrated an area under the curve (AUC) at 0.766. The Youden index established the cut-off at 65.5μm. The patients were divided into two groups depending on the baseline ONL thickness of ≧65.5 μm or not. We compared the association between baseline ONL thickness and the presence of metamorphopsia at 12 months after RFPDT and observed a significant difference between the two groups in the presence of metamorphopsia at 12 months after RFPDT (*P* < 0.001) (Table 7).

**Table 7:** Association between outer nuclear layer thickness at baseline and the presence of metamorphopsia at 12 months.

	Metamorphopsia			
	(+)	(−)	Total	P value*
ONL thickness at baseline				<0.001
≧65.5μm	0	15	15	
<65.5μm	13	8	21	
Total	13	23	36	

ONL, outer nuclear layer.

* Fisher’s exact test.

## Discussion

This study demonstrated that the baseline ONL thickness was associated with metamorphopsia at 12 months after RFPDT in patients with CSC with good baseline visual acuity. Consistent with a previous study [6], no correlation was found between the integrity of the EZ and the severity of metamorphopsia. Bae et al. [10] examined patients with resolved CSC with or without metamorphopsia and found that chronic-recurrent CSC and final disrupted ELM were associated with greater metamorphopsia. The reason for this discrepancy is that all the patients in our study had chronic or recurrent CSC with good baseline visual acuity and that the disruption of the ELM was evaluated within 500 μm from the fovea in our study, whereas Bae et al. [10] evaluated it at 1000 μm from the fovea. Furthermore, vertical and horizontal OCT scans may not reflect the actual ELM or EZ conditions.

In the present study, the duration of symptoms correlated significantly with the M-CHARTS score, which is consistent with the previous report of Bae et al. [10] After multiple regression analysis, we found that the baseline thinner ONL thickness, but not the duration of symptoms, was associated with larger metamorphopsia after RFPDT in patients with CSC with good baseline BCVA. The time of patients’ awareness of symptoms might be later than the onset of actual anatomical change because a slight anatomical change might not affect the visual function of patients.

Ozdemir et al. [11] demonstrated that the ONL thickness at the central fovea was related to symptom duration in patients with CSC and found that the longer the duration of symptom, the thinner the ONL thickness. In their study, the ONL thickness of patients with a symptom duration of less than 1 month was 94.6 ± 10.4 μm, whereas that with a symptom duration of 3–4 months was 70.5 ± 6.7 μm and that with a symptom duration of 4–5 months was 62.7 ± 2.8 μm. The baseline ONL thickness without metamorphopsia was 69.0 ± 16.3 μm in our study. Furthermore, patients with a baseline ONL thickness of ≧65.5 μm did not have metamorphopsia disturbing their daily life. Although the ONL thickness increased after RFPDT, patients should be treated before the ONL thickness drops below 65.5 μm to prevent metamorphopsia after RFPDT. In particular, in recurrent cases, ONL thickness may be reduced with each recurrence. Therefore, considering early intervention of treatment is necessary.

Although patients in our study had good baseline visual acuity, visual acuity was significantly improved after treatment and none of the patients had a logMAR BCVA <0 after treatment. RFPDT may be useful for patients with CSC even with good visual acuity. The ONL thickness was also significantly increased after treatment, consistent with a previous study conducted by Yu J et al., [12] who investigated the relationship between ONL thickness in the active and resolved phases of patients with CSC. Their results demonstrated that the majority of resolved CSC eyes displayed an increase in ONL thickness and the degree of increase was associated with the ratio of the retinal detachment height to the subretinal space width in the active phase. Based on their results, they suggested that retinal stretch contributes to the reduction in ONL thickness. In our study, some patients had increased ONL thickness and some did not, but there was no significant difference between the presence or absence of metamorphopsia after treatment and the amount of ONL thickness increase. Thus, retinal stretch may not be involved in the mechanism of metamorphopsia.

Amsler [13] suggested that the displacement of photoreceptors and the subsequent false localization of the images detected by these photoreceptors result in metamorphopsia. The mechanisms underlying metamorphopsia in patients with CSC remain to be elucidated. Fujita et al. [6] speculated that the regular intervals between adjacent photoreceptors are disrupted by the subretinal fluid in patients with CSC. Another report by Ooto et al. [14] revealed abnormal cone mosaic patterns and reduced cone densities in eyes with resolved CSC. In the present study, the ONL thickness, not the integrity of the ELM or EZ, correlated with metamorphopsia after the resolution of SRD. These results suggest that the loss of photoreceptors due to chronic persistence of SRD causes the displacement of photoreceptors resulting in metamorphopsia, even if not affecting visual acuity.

An important limitation of this study is its relatively small sample size. Moreover, metamorphopsia was investigated at 12 months after treatment, which might be a short follow-up period for evaluating visual prognosis. Therefore, further studies with larger sample sizes and longer follow-up periods are needed.

In conclusion, the baseline ONL thickness was associated with metamorphopsia at 12 months after RFPDT in patients with CSC with good baseline BCVA. In patients with CSC with a certain degree of ONL thickness, considering treatment intervention to protect their quality of vision, irrespective of good baseline BCVA, is necessary.

## Supporting information

S1 Data (XLSX)
